# Editorial: Inorganic chemistry, polymer chemistry, and solid state chemistry editor’s pick 2024

**DOI:** 10.3389/fchem.2025.1562468

**Published:** 2025-01-30

**Authors:** Luís D. Carlos, Pellegrino Musto

**Affiliations:** ^1^ Physics Department and CICECO-Aveiro Institute of Materials, University of Aveiro, Aveiro, Portugal; ^2^ Institute of Polymers, Composites and Biomaterials, National Research Council of Italy, Pozzuoli, Italy

**Keywords:** 1-butene polymerization, antimicrobial properties, MXene-polymer nanocomposites, sonophotocatalysis, radioactive isotopes

We are pleased to introduce the Research Topic Inorganic Chemistry, Polymer Chemistry, and Solid State Chemistry Editor’s Pick 2024, which features thirteen contributions, including ten Original Research articles, two Reviews, and one Perspective, authored by thirty-eight researchers from Algeria, Botswana, China, Iran, Italy, Egypt, Estonia, Ethiopia, France, Germany, Nepal, Pakistan, Saudi Arabia, Taiwan, Turkey, Slovenia, South Africa, Sweden, and the United States. This Research Topic highlights key advancements in experimental, theoretical, and *in silico* research on areas of significant interest for the three sections of the journal, aiming to strengthen the vibrant Frontiers community by recognizing highly meritorious contributions.

As follows, a summary of the scientific contributions is given.

Ali et al. employed advanced exfoliation techniques to produce kaolinite nano-silicate sheets with enhanced adsorption properties, demonstrating a superior surface area and safranin dye uptake, supported by thermodynamic and multi-molecular adsorption analyses.

Hameed et al. synthesized La³⁺-doped Co₀.₅Zn₀.₅LaxFe₂₋_x_O₄ spinel ferrites, demonstrating reduced bandgap and magnetization with increased doping. The materials show potential for microwave and energy storage applications.

Hemmami et al. extracted chitosan from Amanita phalloides, exhibiting excellent antimicrobial properties and water purification potential for pollutants like dyes and drugs, paving the way for eco-friendly applications.

Parajuli reviewed MXene-polymer nanocomposites for biomedical and environmental applications, emphasizing their potential in drug delivery, imaging, and water purification while addressing challenges like biocompatibility.

Khanmiri et al. investigated the leaching behavior of Na and Si in high-level nuclear waste borosilicate glass simulated from a 1000 MWe class PWR reactor, using response surface methodology. Statistical analysis confirmed the significance of pH, contact time, and temperature on leaching, with results consistent with prior studies, validating the applicability of the model.

Hazer et al. synthesized fully biodegradable amphiphilic copolymers from poly (3-hydroxybutyrate) and oligo (2-ethyl oxazoline), showcasing their promising biomedical applications through comprehensive structural characterization.

D’Anania et al. provided mechanistic insights into 1-butene polymerization using DFT simulations, revealing regioselectivity and termination reactions, with implications for designing advanced macromolecular architectures.

Shang uncovered a power-law relationship between neutrons and protons in stable and long-lived radioactive isotopes, offering predictive tools for exploring unknown isotopes.

Esakkimuthu et al. combined molecular dynamics and experimental analysis to study lignin-poly (lactic acid) composites, offering strategies to enhance compatibility by tuning molecular interactions.

Jõul et al. developed lignin-based organic aerogels and porous carbon aerogels, demonstrating tunable morphology and pore structures for tailored applications depending on lignin origin and formulation.

Mapukata et al. reviewed sonophotocatalysis as a promising water treatment technique, highlighting its enhanced efficiency and shorter reaction times. They discussed its mechanisms, commonly used catalysts, and synthesis pathways, along with applications in microbial disinfection and pollutant removal. Challenges, enhancement strategies, and future directions for large-scale implementation were critically analyzed to guide advancements in water treatment technology.

Alem et al. synthesized and characterized two Cr(III) complexes with 1,10-phenanthroline, metformin, and chrysin ligands. The complexes were evaluated for cytotoxicity, antibacterial, and antioxidant activities, with results indicating their potential as promising cytotoxic drug candidates.

Hao et al. synthesized four new U-Te-O compounds under high-temperature, high-pressure conditions: K₂[(UO₂) (Te₂O₇)], Mg [(UO₂) (TeO₃)₂], Sr [(UO₂) (TeO₃)₂], and Sr [(UO₂) (TeO₅)]. These structures exhibit diverse uranium coordination and tellurium oxidation states. Architectures include 1D chains, 2D layers, and 3D frameworks, featuring edge-sharing polyhedra and tunnels formed by 6-membered rings. The study highlights the structural flexibility and synthetic potential of the U-Te-O system.

This Research Topic underscores the interdisciplinary nature of contemporary research and celebrates the diverse contributions advancing our understanding of these important fields.

